# Yarrow Supercritical Extract Ameliorates the Metabolic Stress in a Model of Obesity Induced by High-Fat Diet

**DOI:** 10.3390/nu12010072

**Published:** 2019-12-26

**Authors:** Lamia Mouhid, Marta Gómez de Cedrón, Adriana Quijada-Freire, Pablo J. Fernández-Marcos, Guillermo Reglero, Tiziana Fornari, Ana Ramírez de Molina

**Affiliations:** 1 Molecular Oncology and Nutritional Genomics of Cancer, IMDEA-Food Institute, CEI UAM + CSIC, 28049 Madrid, Spain; Lamia.Mouhid@imdea.org (L.M.); Adriana.quijada@imdea.org (A.Q.-F.); guillermo.reglero@imdea.org (G.R.); 2 Metabolic Syndrome Group, IMDEA-Food Institute, CEI UAM + CSIC, 28049 Madrid, Spain; pablojose.fernandez@imdea.org; 3 Production and Characterization of Novel Foods Department, Institute of Food Science Research (CIAL) CEI UAM + CSIC, 28049 Madrid, Spain; tiziana.fornari@uam.es

**Keywords:** yarrow supercritical extract, obesity, diabetes, insulin resistance, hypercholesterolemia, fatty liver, cancer

## Abstract

Nowadays, obesity and its associated metabolic disorders, including diabetes, metabolic syndrome, cardiovascular disease, or cancer, continue to be a health epidemic in westernized societies, and there is an increased necessity to explore anti-obesity therapies including pharmaceutical and nutraceutical compounds. Considerable attention has been placed on the identification of bioactive compounds from natural sources to manage the metabolic stress associated with obesity. In a previous work, we have demonstrated that a CO_2_ supercritical fluid extract from yarrow (Yarrow SFE), downregulates the expression of the lipogenic master regulator *SREBF1* and its downstream molecular targets *FASN* and *SCD* in a tumoral context. Since obesity and diabetes are strongly considered high-risk factors for cancer development, herein, we aimed to investigate the potential therapeutic role of Yarrow SFE in the metabolic stress induced after a high-fat diet in mice. For this purpose, 32 C57BL/6 mice were distributed in four groups according to their diets: standard diet (SD); SD supplemented with Yarrow SFE (SD + Yarrow); high-fat diet (HFD); and HFD supplemented with Yarrow SFE (HFD + Yarrow). Fasting glycemia, insulin levels, homeostasis model assessment for insulin resistance (HOMA-IR), lipid profile, gene expression, and lipid content of liver and adipose tissues were analyzed after three months of treatment. Results indicate improved fasting glucose levels in plasma, enhanced insulin sensitivity, and diminished hypercholesterolemia in the HFD + Yarrow group compared to the HFD group. Mechanistically, Yarrow SFE protects liver from steatosis after the HFD challenge by augmenting the adipose tissue buffering capacity of the circulating plasma glucose.

## 1. Introduction

According to the World Health Organization (WHO), in 2016, more than 650 million people were estimated as obese. The prevalence of obesity and its associated metabolic disorders, including diabetes, metabolic syndrome, cardiovascular disease, and cancer is rapidly becoming a severe global health problem [1,2].

In the course of obesity, the increased lipid accumulation induces a systemic chronic inflammation that promotes an abnormal cellular response to insulin, leading to insulin resistance and type 2 diabetes (T2D). In this regard, the International Federation Atlas (2018) has estimated over 415 million people diagnosed with diabetes, with 90% of the cases being Type 2 diabetes (T2D) [3,4,5]. Whether insulin resistance triggers hyperinsulinemia or hyperinsulinemia, in turn, causes insulin resistance is still under debate [6].

Obesity and type 2 diabetes (T2D) not only are considered risk factors for the appearance of multiple types of cancers (liver, breast, colon, pancreatic, esophageal, among others), but also correlate with poorer prognosis [2,3,4,5,6,7,8]. WHO has estimated that one-third of cancers could be prevented by modifying risk factors, such as augmenting physical activity and reducing the intake of saturated fatty acids or high-glucose-containing drinks.

Current therapeutic strategies for attenuating obesity and insulin resistance include lifestyle modifications (e.g., diet, exercise, weight loss) prior to the administration of pharmacological agents (e.g., insulin-sensitizing drugs), which are prescribed only for patients with a body mass index (BMI) ≥27 kg/m^2^. Bariatric surgery is considered only for patients with a BMI of 30–40 kg/m^2^ [9,10]. Due to the complications of insulin-sensitizer drugs, alternative remedies in the form of dietary agents to attenuate insulin resistance are receiving more interest.

In this regard, phytochemicals from natural sources are drawing attention, as therapeutic or preventing agents to manage alterations associated with chronic diseases related to metabolism [11,12]. Some of them have been shown to reduce adipose tissue mass, to decrease metabolic stress, to mimic the action of insulin and/or to act as potent anti-hyperglycemic agents [13,14,15].

In a previous work, we have demonstrated that Yarrow (*Achillea millefolium*) supercritical fluid extract (Yarrow SFE) downregulates the expression of the lipogenic genes *SREBF1*, *FASN*, and *SCD1* in a pancreatic cancer model [16]. Herein, with obesity and diabetes being considered risk factors in cancer [17], we aimed to investigate whether Yarrow SFE could restore the lipid and/or glucose homeostasis after a high-fat diet induced obesity.

Our results indicate Yarrow SFE improves circulating fasting glucose levels, enhances insulin sensitivity, and diminishes hypertriglyceridemia and hypercholesterolemia. Mechanistically, Yarrow SFE protects liver from steatosis after the high-fat diet (HFD) challenge by augmenting the adipose tissue buffering capacity of the circulating plasma glucose. In conclusion, Yarrow SFE could be proposed as a nutritional supplement in obesity and diabetes therapy.

## 2. Materials and Methods

### 2.1. Yarrow Supercritical Fluid Extract (SFE)

Yarrow SFE extract was obtained with supercritical CO_2_ using the extractor Thar Technology model SF2000 (Pittsburgh, PA, USA), with a 2 L extraction cylinder and two 0.5 L separators. Pressure (±0.1 MPa) and temperature (±2 K) were controlled independently.

Ground Yarrow was subjected to 40 °C and 140 Bar with a CO_2_ flux of 70 g/min during 180 min.

The composition and chemical characterization of this extract has been described previously [18].

### 2.2. Animals and Treatment

In this study, C57BL/6 mice were housed in a temperature- and humidity-controlled room with a cycle of 12 h light/darkness allowing free access to water and food. A total of 32 mice were randomly distributed in two groups according to their diets (values are referenced to the percentage of the energy they contribute—Kcal/kg): standard chow diet (SD) group (proteins 18.2%, carbohydrates 73.1%, and fat 8.6%; D40 -Rats, Mice Maintenance Diet-RMM, Safe) and high-fat diet group (HFD) (proteins 20%, carbohydrates 35%, and fats 45%; D12451 Research Diets, Inc., New Brunswick, NJ, USA). After three months with the SD or the HFD, animals of each group were randomly distributed in two additional groups for the intervention period (in the presence or absence of Yarrow SFE, 800 mg/Kg Yarrow five times per week) during three months ending up with four groups: SD; SD + Yarrow; HFD; and HFD + Yarrow. The dose was chosen based on a previous study to determine the putative toxicity of the extract in acute. Different doses ranging from 200 to 1000 mg/Kg of Yarrow SFE were administered to mice by gavage. No effects were observed on markers regardless of renal or liver dysfunctions or the animal’s general health or behavior.

All procedures of the study were approved by the Research and Animal Welfare Ethics Committee of the Consejo Superior de Investigaciones Cientificas (CSIC) and the Environment Counseling of Community of Madrid (PROEX112/17), under the provisions of RD53/2013 law. The experiments were performed in the animal facilities of the Center for Biological Research (CIB-CSIC).

Animals were anesthetized with isoflurane 4–5%, followed by a cardiac puncture, in order to collect the maximum volume of blood.

### 2.3. Glucose Tolerance Test and Insulin Level Measurement

Glucose tolerance tests (GTT) were performed on mice fasted for 18 h. In all cases, 2 g/kg of glucose (Sigma) was intraperitoneally injected. Blood glucose levels were measured in fasted animals at 15, 30, 60, and 120 min after the glucose injection by mean of the Glucomen blood glucose monitoring system (A. Menarini Diagnostics).

Insulin levels in fasted animals were measured with the Ultrasensitive Mouse Insulin ELISA (enzyme linked immunosorbent assay) kit (#90080 Crystal Chem.).

The HOMA-IR index (homeostasis model Assessment for insulin resistance) was calculated with the formula (insulin × glucose/22.5), where insulin and glucose are the fasting insulin and glucose levels [19].

For GTT and insulin tolerance test (ITT) analysis, blood was obtained after a superficial cut at the end of the tails. For insulin determinations, the same protocol was used but with tubes designed to be filled by capillarity, which allowed to obtain up to 20 μL.

### 2.4. Biochemical Analysis

Body weight was monitored twice a week and food intake once per week.

The entire blood, obtained by the cardiac puncture at the end of the study, was used to determine the levels of the (i) hepatic ALT (alanine transaminase) and AST (aspartate transaminase) enzymes, (ii) the lipid profile (total cholesterol, HDL-cholesterol, LDL-cholesterol, NEFA (non-esterified fatty acids), and (iii) the urea levels.

All the biochemical determinations were carried out at the Center for Applied Medical Research of Navarra. Serum levels of cholesterol, ALT, AST, urea, and NEFAs were determined using a Cobas C311 Autoanalyzer (Roche, Basel, Switzerland) by routine laboratory methods from Roche or, in the case of NEFAs, from Wako (Wako Chemicals Europe, Neuss, Germany).

### 2.5. RT-PCR and Real-Time PCR

Liver and adipose tissues obtained from the sacrificed animals at the end of the study, were immediately frozen. RNA extraction from tissues was performed in dried ice to minimize RNA degradation. Samples were cut and quickly introduced in Trizol (Qiagen, Madrid, Spain) followed by a homogenization with the Ultra-Turrax (T10, IKA, Breisgau, Germany). RNA was extracted using the miRNeasy Mini Kit (Qiagen, Hilden, Germany), which combines cell lysis with a silica membrane-based purification. Total RNA was quantified with UV–vis spectrophotometer (NanoDrop 2000/2000c, ThermoFisher, Madrid, Spain). Reverse transcription was performed with the High-Capacity cDNA Reverse Transcription kit (ThermoFisher, Madrid, Spain), following manufacturer’s instructions. RT-PCR reactions were performed in 20 µL at 25 °C 10 min, 37 °C 120 min, and 85 °C 5 min. The 7900HT Fast Real-Time q-PCR (Applied Biosystems, Waltham, MA, USA) was done using the same amount of cDNA of each tissue, with specific probes (Taqman): *SREBF1* (Mm00550338_m1); *FASN* (Mm00662319_m1), *SCD* (Mm00772290_m1), *GLUT4* (Mm00436615_m1), *IRS1* (Mm01278327_m1), and two endogenous house-keeping genes: *18S* (Mm03928990_ g1) and *GAPDH* (Mm99999915_g1). Gene expression analysis was carried out with the software RQ Manager (ThermoFisher, Madrid, Spain), and the relative expression (RQ) of each gene was determined following the 2^−∆∆^*^C^*^t^ method (Livak) [20].

### 2.6. ^1^H NMR Analysis of the Lipid Content in Liver and Adipose Tissues, and Glycogen and Choline Content in Liver Tissues

High Resolution Magic Angle Spinning (HR MAS) spectra were acquired from an 11.7 Tesla Bruker Avance spectrometer operating at 500.113 MHz, at 4 °C and 5 KHz spinning rate. 1D ^1^H HR MAS spectra were acquired using Carr–Purcell–Meiboom–Gill (CPMG) sequence with 2 s water pre-saturation, 144 ms echo time, and 128 scans. Data were collected into a 32 K data point using a spectral width of 10 KH (20 ppm) and water pre-saturation during a relaxation delay of 2 s and 1D sequence for the diffusion measurement using stimulated echo with bipolar gradient pulses (stebpgp1s1d) with big delta 200 ms and little delta 1.2 ms. A sine-shaped gradient was followed by a 300 μs delay for gradient recovery, 5 kHz spectral width, 32 K data point, and 128 scans. Quantification of detectable metabolites in the ex vivo spectra was performed by measuring the area of the peaks using the MestReC software (Mestrelab Research, Santiago de Compostela, Spain). Data were manually phased and baseline corrected. NMR spectra were referenced to the FA terminal –CH_3_ signal at δ 0.89 ppm. The analysis of the metabolites was performed by selecting the following resonances:

⋅ Methyl groups (–CH_3_) at 0.89 ppm (saturated fatty acid chains)
⋅ –CH_3_ at 0.96 ppm (n-3 EPA and DHA)
⋅ Acyl chains methylenes ((CH_2_)_n_ at 1.33 ppm
⋅ CH_2_–C–CO at 1.58 ppm
⋅ CH_2_C=C at 2.02 ppm
⋅ CH_2_CO other than DHA at 2.25 ppm
⋅ CH_2_CO of DHA at 2.33 ppm
⋅ =C–CH_2_–C= at 2.78 ppm
⋅ –CH=CH– at 5.33 ppm
⋅ Choline at 3.2 ppm
⋅ Phosphocholine at 3.19 ppm
⋅ Glycogen at 5.3 ppm

### 2.7. Statistical Analysis

Results are shown as means ± the standard error of the mean (SEM). Statistical analyses were performed using GraphPad (v.7.03 Software, Inc., San Diego, CA, USA). The comparison between the groups was performed by one-way ANOVA followed by Bonferroni’s post-test. Statistical differences were considered as follows: * *p* ≤ 0.05; ** *p* ≤ 0.01; *** *p* ≤ 0.001; **** *p* < 0.0001.

## 3. Results

### 3.1. Study Workflow

As Yarrow SFE diminishes the expression of *SREBF1* in pancreatic cancer cells and in a xenograph mouse model [16], we wanted to analyze the impact of Yarrow SFE in C57/Bl6 mice under standard diet (SD) or high-fat diet (HFD)-induced obesity.

Two different glucose and insulin tolerance tests were performed: (1) after three months of intervention with SD or HFD, in the absence of Yarrow, and (2) after three additional months in which the SD or HFD was combined or not with Yarrow (final end point of the experiment was six months).

The time elapsed between the end of the second test and the sacrifice of animals was one week to allow animals to recover from the GTT/ITT intervention.

**Figure 1:**
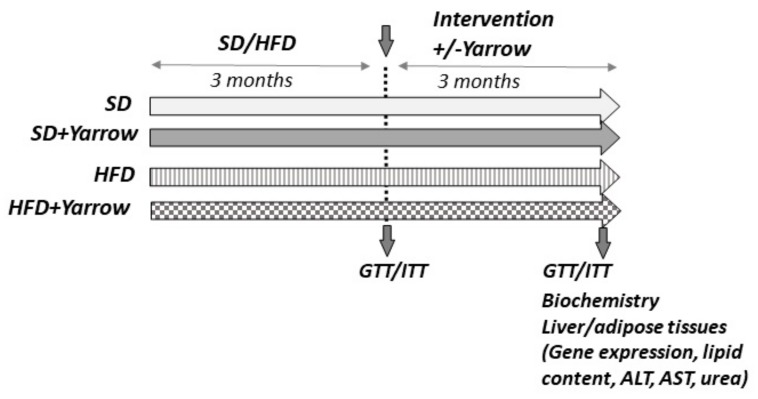
Study workflow together with the main biochemical and molecular determinations. SD—standard diet; HFD—high-fat diet; GTT—glucose tolerance test; ITT—insulin tolerance test—ALT—alanine transaminase; AST—aspartate transaminase.

Figure 1 shows the study workflow together with the biochemical and molecular determinations.

### 3.2. Yarrow SFE Diminishes Plasma Glucose Levels in a Pre-Diabetic Model Induced by HFD

After three months of SD or HFD, a glucose tolerance test (GTT) was performed to confirm the pre-diabetic state in the HFD group compared to SD group.

**Figure 2:**
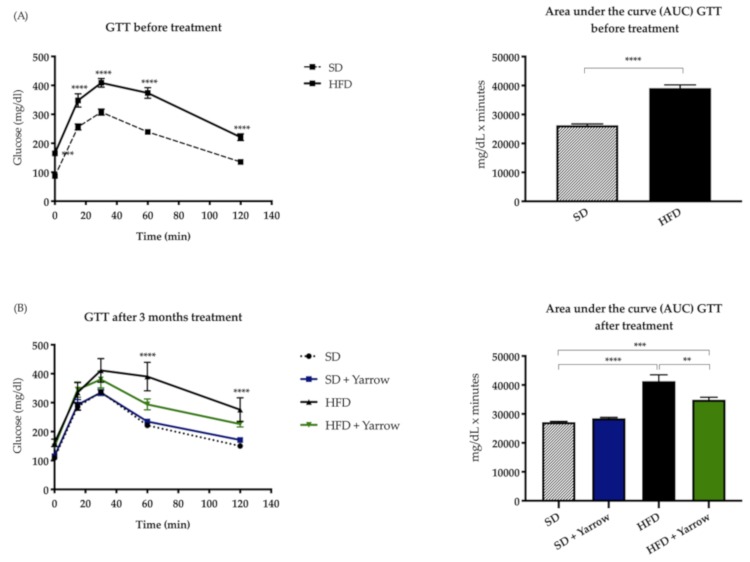
(**A**) GTT test and area under the curve (AUC) comparisons between SD and HFD groups showing statistically significant differences between the SD and HFD groups (pre-diabetic state). (**B**) GTT test and the AUC comparisons between SD ± Yarrow and HFD ± Yarrow after the three additional months of the intervention period (yarrow treatment was 800 mg/kg). Values are means ± SEM of 6–8 mice per condition. ** *p* < 0.01; *** *p* < 0.001; **** *p* < 0.00001.

As shown in Figure 2A, mice exposed to HFD, displayed statistically significant higher circulating glucose levels compared to the SD group. These results indicate hyperglycemia and a pre-diabetic state in the HFD group.

At this point, the intervention period—absence/presence of Yarrow for three additional months—was initiated, and after this a second GTT analysis was performed. There were not found statistically significant differences between the SD and the SD + Yarrow groups, indicating that yarrow had no impact on the circulating glucose levels under SD. As expected, the HFD group displayed increased circulating levels of glucose compared to the SD group. Importantly, only the HFD + Yarrow group restored the levels of circulating glucose to that of the SD group after 60 min of the glucose injection (Figure 2B). These results indicated that Yarrow SFE was able to ameliorate, to some extent, the glucose levels in the obese and hyperglycemic animals.

### 3.3. Yarrow SFE Improves Insulin Sensitivity in a Pre-Diabetic Model Induced by HFD

**Figure 3:**
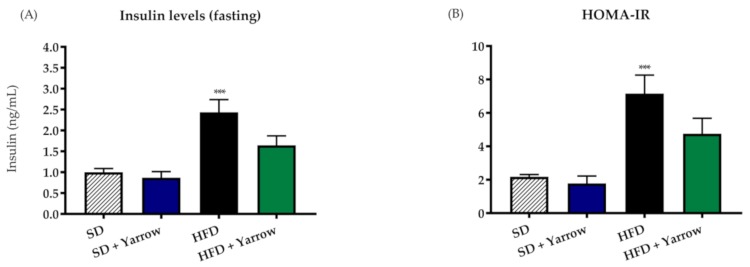
Insulin levels (**A**) and homeostasis model assessment for insulin resistance (HOMA–IR) (**B**) after three months of the intervention period ± Yarrow (800 mg/kg). Values are expressed as means ± SEM, 6–8 mice per condition. Asterisks indicate differences related to the SD group. *** *p* < 0.001.

To study the impact of Yarrow SFE in the sensitivity to insulin, basal insulin levels were quantified in the four groups after 18 h of fasting. As expected, HFD displayed statistically significant increased insulin levels compared to the control SD group. Importantly, insulin levels in the HFD + Yarrow group was not statistically different from that of the SD group (Figure 3A).

Moreover, calculated HOMA-IR index, which estimates the resistance to insulin, showed higher index in the HFD group compared to the HDF + Yarrow group (Figure 3B). Thus, animals treated with yarrow, although still considered as insulin resistant (IR > 3), displayed an improved sensitivity to insulin.

The food intake was monitored (once per week) and so was the body weight (twice per week). No significant differences were found in the amount of food intake compared to SD group (Figure S1). Regardless of body weight, no significant differences were found between the HFD + Yarrow and HFD groups, nor between the SD + Yarrow and the SD groups in our experimental conditions (Figure S2).

### 3.4. Yarrow SFE Decreases Total Plasma Cholesterol Levels and NEFA Levels after HFD-Induced Obesity

**Figure 4:**
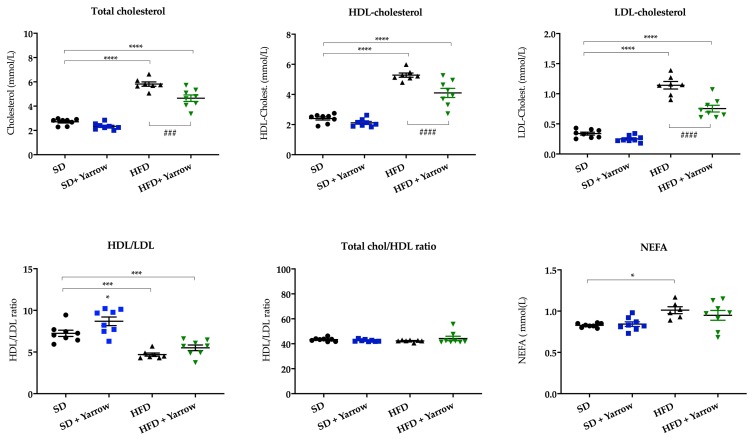
Lipid prolife of SD and HFD mice treated or not with Yarrow SFE (supercritical fluid extract). Values represent the mean ± SEM of 6–8 mice per condition. Statistical differences relative to the SD group are indicated as * *p* < 0.05; *** *p* < 0.001 and **** *p* < 0.0001. Statistical differences found between HFD and HFD Yarrow groups are indicated as ### *p* < 0.001 and #### *p* < 0.0001.

As obesity is frequently associated with increased circulating plasma levels of NEFA and cholesterol, we wanted to evaluate the impact of Yarrow SFE on these parameters. As shown in Figure 4, NEFA diminished in the HFD + Yarrow group compared to the HFD group. Similarly, total cholesterol levels were improved in the HFD + Yarrow group compared to the HFD group, although without recovering to the levels of the SD groups. In addition, the ratio LDL-C/HDL-C was also improved in the HFD + Yarrow group compared to the HFD group.

### 3.5. Yarrow SFE Is Well Tolerated and Diminishes Biomarkers of Hepatic Damage

**Figure 5:**
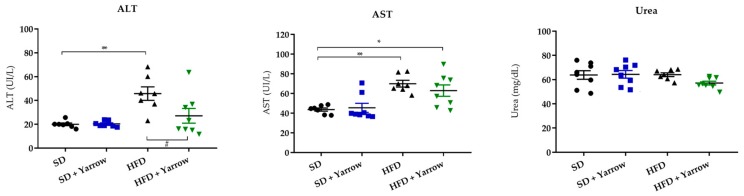
Analysis of circulating hepatic and renal parameters (ALT, AST, Urea) of obese and normal mice treated with 800 mg/kg Yarrow SFE extract and their controls. Values represent the mean ± SEM of 6–8 mice per condition. * Indicates differences compared to the SD group; and # to the HFD group. * *p* < 0.05; ** *p* < 0.01.

To discard any toxicity associated with the administration of Yarrow SFE after a prolonged administration (three months of intervention period), biomarkers of the hepatic and renal functions were analyzed: ALT, AST, and urea levels. As shown in Figure 5, AST and ALT levels were found increased in the HFD group compared to the SD group. Importantly, ALT circulating levels were statistically diminished in the HFD + Yarrow group compared to the HFD group. The AST circulating levels displayed a tendency to diminish, although without reaching the levels of the control SD group. Regarding the urea levels, all groups behaved similarly. These results indicated that Yarrow SFE was well tolerated.

### 3.6. Yarrow SFE Reduces De Novo Lipogenesis and the Fatty Acid Content in Livers after HFD-Induced Obesity

**Figure 6:**
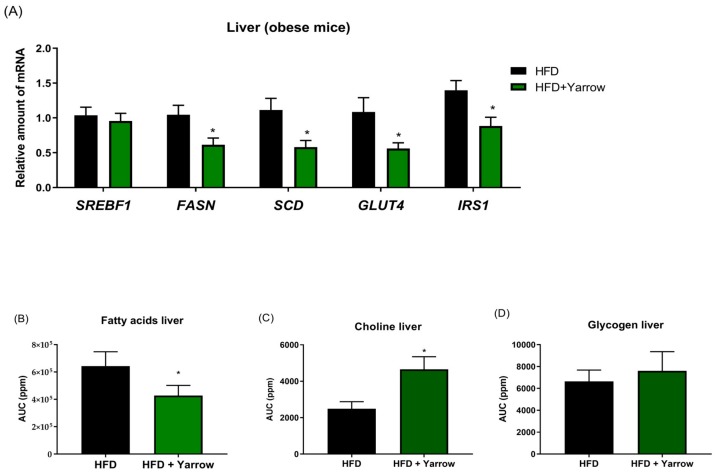
(**A**) Gene expression of *SREBF1, FASN*, *SCD1*, *GLUT4*, and *IRS1* genes in livers from HFD + Yarrow and HFD groups. (**B**) Quantification of the relative fatty acid content. (**C**) Choline and (**D**) glycogen contents of the livers from HFD + Yarrow and HFD groups (NMR analysis of 10 mg of tissues). Data represent the mean ± SEM of 6–8 mice per condition. * *p* < 0.05.

To study the effect of Yarrow on the hepatic de novo lipogenesis, we first quantified the expression levels of the master lipogenic transcription factor *SREBF1*, and its downstream molecular targets *FASN* and *SCD1*. As shown in Figure 6A, the HFD + Yarrow group displayed a statistically significant reduction in the expression levels of *FASN* and *SCD1* compared to the HFD group. 

As the liver is a crucial organ for the maintenance of the systemic glucose homeostasis, we also analyzed the expression levels of the glucose transporter (*GLUT4*) and the insulin receptor substrate 1 (*IRS1*), a downstream regulator of the insulin signaling. As shown in Figure 6A, the *GLUT4* and *IRS1* expression levels were diminished in the HFD + Yarrow group compared to the HFD group, although without restoring the levels to that of the SD animals (not shown). These results indicate that livers from the HFD + Yarrow group had a diminished insulin signaling compared to the HFD group which, together with the improved HOMA-IR, reinforces a role of Yarrow SFE in alleviating insulin resistance.

By means of NMR analysis, we quantified the relative fatty acid content and choline and glycogen levels of livers from the HFD + Yarrow and the HFD groups (10 mg of tissue analyzed). As shown in Figure 6B, the relative comparison of the fatty acid liver contents showed a tendency to diminish in the HFD + Yarrow group, although not statistically significant. Importantly, the relative choline levels (choline and phosphate choline) were statistically improved in livers from mice in the HFD + Yarrow group compared to the HFD group (Figure 6C). Regardless of glycogen content no statistically significant differences were found between HFD + Yarrow and HFD (Figure 6D).

In summary, all these results suggest a better performance of livers from the HFD + Yarrow group compared to those from the HFD group.

### 3.7. Yarrow SFE Increases the Uptake of Glucose in the Adipose Tissue but Not the Adipose Tissue Fatty Acid Content

Adipose tissue (AT), which is insulin-sensitive, is considered a key regulator of the systemic metabolism and its deregulation encompasses insulin resistance, dyslipidemia, and/or glucose intolerance [21,22].

In a non-obese situation, after feeding, insulin inhibits the AT lipolysis promoting lipogenesis. On the contrary, during fasting or exercising, lipolysis is induced. In this way, AT acts as an energy buffer to store the excess of energy and to prevent harmful effects of FFA elsewhere in the body. Nevertheless, during insulin resistance, AT can promote the ectopic deposition of fatty acids or lipotoxicity in other tissues.

As Yarrow SFE improved the insulin sensitivity and the plasma lipid profile, we wanted to know whether Yarrow SFE could affect the glucose uptake (*GLUT4*, *IRS1*) and the TG storage capacity (*SREBF1*, *FASN*, *SCD1*) of the AT after the HFD challenge.

**Figure 7:**
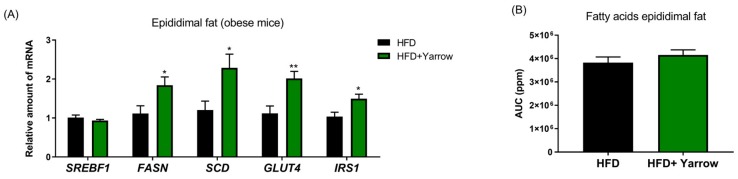
(**A**) Gene expression of the lipogenic *SREBF1*, *FASN*, and *SCD1* genes and *Glut4* and *IRS1* genes of epididymal adipose tissues from HFD + Yarrow and HFD groups. (**B**) Quantification of relative fatty acid content of epididymal adipose tissues from HFD + Yarrow and HFD groups (NMR analysis comparison from 10 mg of tissue). Data represent the mean ± SEM of 6–8 mice per condition. * *p* < 0.05, ** *p* ≤ 0.01.

As observed in Figure 7A, the HFD + Yarrow group significantly increased the expression levels of *FASN*, *SCD1*, *Glut4*, and *IRS1* compared to the HFD group. These results suggest an improved insulin sensitivity of the AT favoring the uptake of circulating glucose to be redirected toward the synthesis of FAs with more control of unsaturated FAs (Figure 7B). Importantly, the expression levels of *SREBF1* were not augmented, and the relative comparison of the fatty acid content was similar to the HFD + Yarrow group (NMR analysis). This, together with the reduced circulating levels of FFA, suggests that Yarrow improves the systemic metabolic stress after the HFD challenge.

## 4. Discussion

The main objective of the present work was to assess the potential benefit of Yarrow SFE extract in a model of high-fat diet induced obesity. For this purpose, and as indicated in the study workflow (Figure 1), 32 mice were distributed in two groups. After three months of SD (n = 16) or HFD (n = 16), the GTT analysis indicated a pre-diabetic status in the HFD group. The subsequent three months of the intervention period (presence or absence of Yarrow SFE) showed that Yarrow SFE was able to ameliorate the hyperglycemia compared to the HFD group. No differences were found between mice in the SD ± Yarrow groups (Figure 2).

In addition, insulin levels were improved in the HFD + Yarrow group compared to the HFD group (Figure 3).

Yarrow SFE was able to alleviate to some extent the lipid profile in plasma and the hepatic lipid accumulation when compared to the HFD group (Figure 4). Moreover, Yarrow SFE diminished biomarkers of hepatic damage in the HFD + Yarrow group compared to the HFD group (Figure 6).

Different mechanisms seem to be responsible for these effects.

Insulin is a key regulator of many processes. In normal conditions, in the adipose tissue, insulin promotes glucose to be re-directed toward de novo lipogenesis and triglyceride storage. On the other hand, in the liver, insulin stimulates the synthesis of glycogen and diminishes the hepatic gluconeogenesis, preventing the efflux of glucose into the bloodstream. Nevertheless, during obesity and diabetes, insulin resistance is manifested by increased lipolysis from adipose tissue and increased de novo lipogenesis and gluconeogenesis in the liver [23,24,25].

In this study, Yarrow SFE influences the glucose and lipid homeostasis between liver and adipose tissues. Livers from the HFD + Yarrow group displayed a significant decrease in the expression levels of *FASN* compared to the HFD group. Moreover, the decreased in the expression levels of *IRS1* suggests a decreased intracellular insulin signaling in livers from the HFD + Yarrow group. This is in accordance with the NMR quantification of the fatty acid liver content, which showed a tendency toward a reduction of the total fatty acid content. It would have been interesting to determine the intrahepatic cholesterol levels, although the improvement in the choline levels suggests a better performance of livers from the HFD + Yarrow group compared to the HFD group.

During obesity and insulin resistance, the adipose tissue presents (i) a diminished ability to capture glucose due to the downregulation of *GLUT4*, the major insulin-regulated glucose transporter [26,27], and (ii) an increased lipolysis and release of fatty acids and glycerol [28,29], shifting down the lipogenic process.

Adipose tissue has developed adaptive mechanisms to cope with the metabolic stress during obesity. Herein, we found that although the expression levels of *SREBF1* were kept similar, *FASN* and *SCD1* expression levels were increased in the HFD + Yarrow group compared to the HFD group. Importantly, Yarrow SFE increased the expression levels of the glucose transporter *GLUT4*. This, together with the increased *FASN* and *SCD1* expression levels in the adipose tissue, suggests an increased capability of adipose tissues from HFD + Yarrow group to redirect the circulating glucose toward the synthesis of fatty acids and triglycerides. The increase in *GLUT4* may also partially explain the diminished hyperglycemia found in the HFD + Yarrow group compared to the HFD group.

There is controversy regarding whether the increase of *FASN* and *SCD1* in the adipose tissue of obese individuals is or is not an SREBP1-dependent process [30]. In the last years, many results support the idea that *SREBF1* is not responsible of the increased lipogenesis in the adipose tissue [31] due to the activation of *FASN* and *SCD*1 regulated by the intracellular glucose levels. The regulatory feedback control point of the *Insig1/SREBP1* axis allows to compensate the anti-lipogenic effects associated with insulin resistance by augmenting the *FASN* and *SCD1* expression levels without augmenting the expression levels of *SREBF1*, although regulating the protein levels of the mature SREBP1 transcription factor [32]. Here, *SREBF1* expression levels were not increased in the epididymal adipose tissue of the HFD + Yarrow group compared to the HFD group, suggesting that the increase of *FASN* and *SCD1* may be regulated by the increase glucose uptake. Similar results have been found in vitro in 3T3L1 cells treated with Yarrow SFE (data not shown).

No significant differences were found between body weights of HFD + Yarrow and HFD groups, nor between SD + Yarrow and SD groups in our experimental conditions. Although in this study the subcutaneous adipose tissue was not quantified, the observed reduction of *FASN* and *SCD*1 in the livers of the HFD + Yarrow group, together with the reduction of hepatic damage biomarkers, suggests a healthier distribution of adipose tissue against visceral lipotoxic accumulation.

## 5. Conclusions

In the course of obesity and insulin resistance, increased lipolysis from adipose tissue is observed. The excess of TG and NEFA in ectopic tissues such as liver, pancreas, and muscle, disrupts the systemic metabolic control of lipids and glucose. Insulin resistance in liver, induces gluconeogenesis and de novo fatty acid synthesis, ending up with increased glucose release and hepatic steatosis [33,34].

Herein, Yarrow SFE improved the plasma glucose levels and ameliorated the insulin resistance in an HFD model of obesity. Yarrow SFE augmented the adipose tissue buffering capacity of circulating glucose toward the synthesis of fatty acids and triglycerides. In addition, Yarrow SFE diminished the expression levels of *FASN* and *SCD1* in livers after an HFD, reducing the overall fatty acid liver content. Importantly, Yarrow SFE also diminished the expression levels of *IRS1* in the livers which may contribute to decrease the intracellular insulin signaling after an HFD. The increased in the choline levels suggests a better performance of livers from the HFD + Yarrow group compared to the HFD group, as high choline levels have been shown to protect against fatty liver disease or even hepatocarcinoma [35,36]. Linked to this, the HFD + Yarrow group presented a tendency toward increased levels of glycogen which suggests a better performance for the storage of glucose in the liver in accordance with the improvement in insulin sensitivity.

Although an ethanolic extract of Yarrow has been described to improve the circulating glucose levels in a diabetic model [37], herein SFE Yarrow also improved the lipid profile and ameliorated the hepatic steatosis in an HFD model of obesity. Altogether, our results indicate that Yarrow SFE may be beneficial for obese and hypercholesterolemic patients.

## Supplementary Materials

The following are available online at https://www.mdpi.com/2072-6643/12/1/72/s1, Figure S1: Food intake monitoring of SD and SD + Yarrow groups, and HFD and HFD +Yarrow groups. No significant differences were found among groups compared to control SD group. Figure S2: Body weight monitoring of SD and SD + Yarrow groups, and HFD and HFD +Yarrow groups. No significant differences were found between HFD + Yarrow and HFD groups, nor between SD + Yarrow and SD.
