# Supplementary figures and images for: Yarrow Supercritical Extract Ameliorates the Metabolic Stress in a Model of Obesity Induced by High-Fat Diet
Source: Nutrients. 2019 Dec 26;12(1):72. doi: 10.3390/nu12010072 (PMC7019615; doi:10.3390/nu12010072)

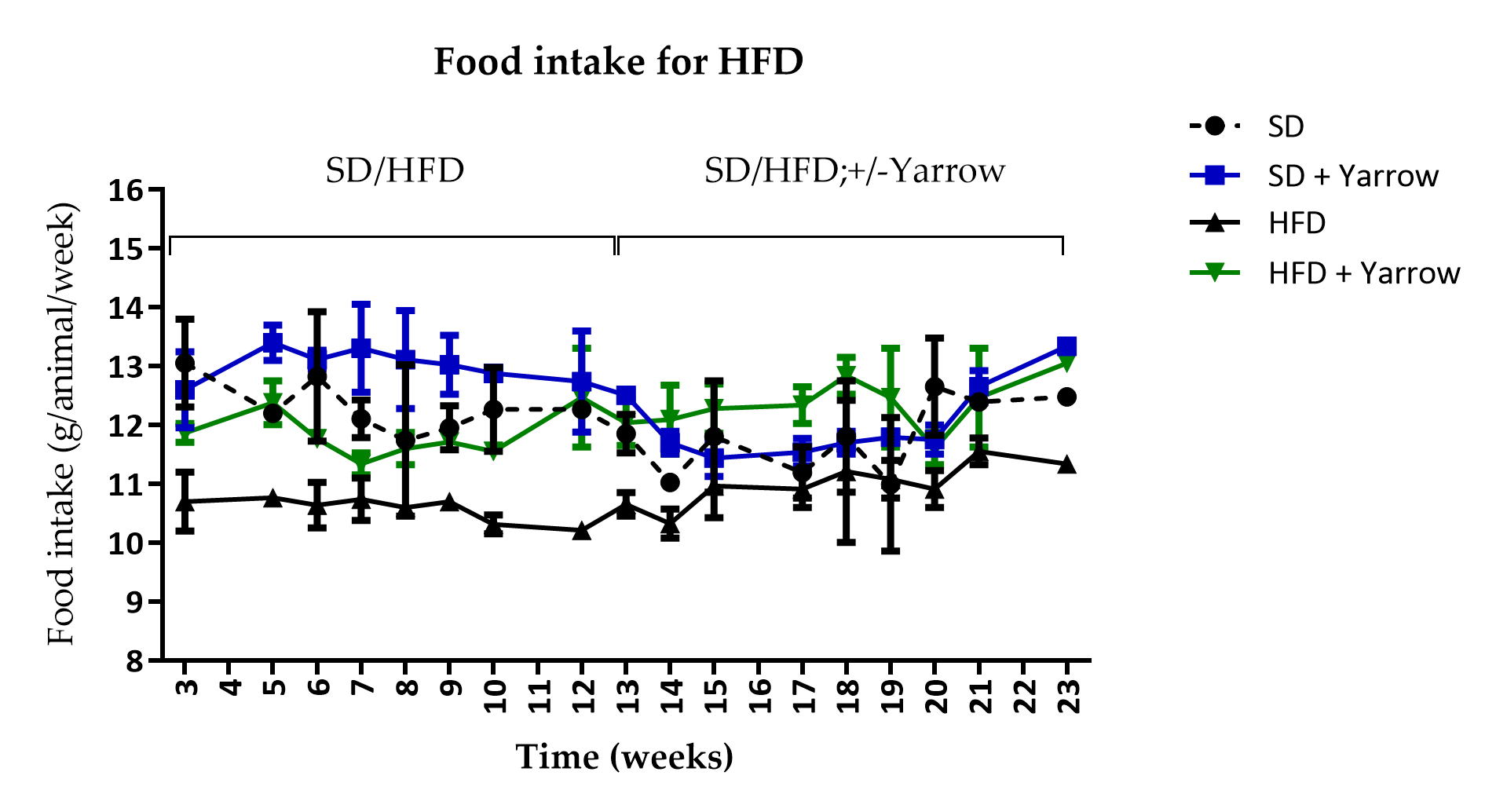

Supplement: Supplementary file 1 [file nutrients-12-00072-s001.zip › Figure S1.tif]

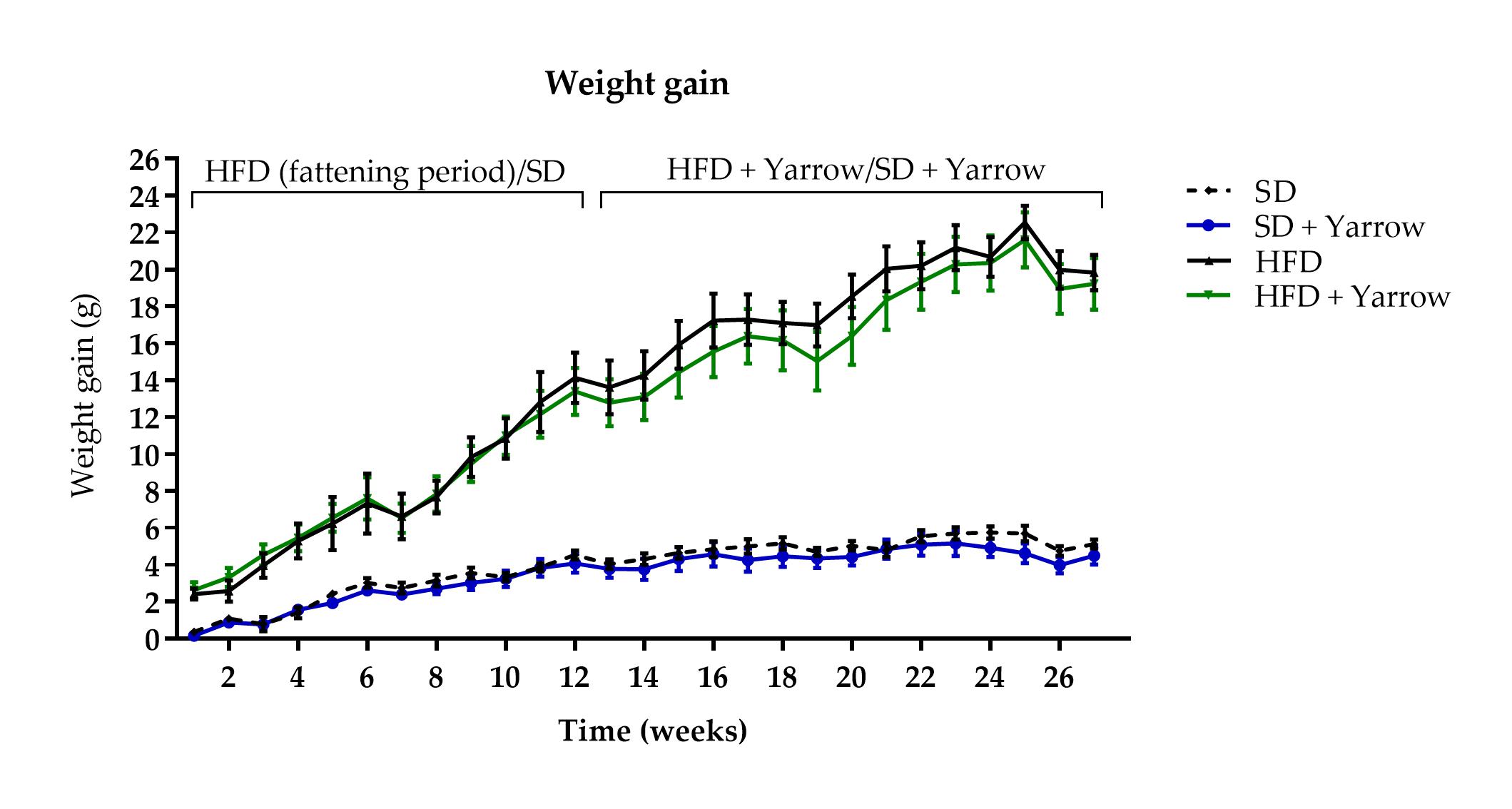

Supplement: Supplementary file 1 [file nutrients-12-00072-s001.zip › Figure S2.jpg]
